# Skeletal muscle and abdominal circumference explain intramuscular fat, independent of exercise frequency, in middle-aged Japanese men

**DOI:** 10.1371/journal.pone.0267557

**Published:** 2022-05-25

**Authors:** Hiroshi Akima, Hisashi Maeda, Masataka Suwa, Takayuki Imoto, Noriko Tanaka

**Affiliations:** 1 Research Center of Health, Physical Fitness & Sports, Nagoya University, Nagoya, Aichi, Japan; 2 Graduate School of Education & Human Development, Nagoya University, Nagoya, Aichi, Japan; 3 Graduate School of Medicine, Nagoya University, Nagoya, Aichi, Japan; 4 Health Support Center WELPO, Toyota Motor Corporation, Iwakura, Toyota, Aichi, Japan; Ritsumeikan University, JAPAN

## Abstract

The purpose of this study was to examine how intramuscular adipose tissue (IntraMAT) can be characterized using physical and functional characteristics, muscle size, and/or adipose tissue in four different exercise frequency groups of middle-aged Japanese men. One thousand twenty-eight middle-aged men (age, 47.5 ± 8.1 years; height, 170.5 ± 5.8 cm; body mass, 67.0 ± 9.3 kg; body mass index, 21.9 ± 5.3 kg/m^2^) were allocated to four groups on the basis of their weekly exercise frequency: Group 1, no exercise, n = 334; Group 2, once a week, n = 271; Group 3, a few days a week, n = 269; and Group 4, every day, n = 154. Their body composition, blood pressure, and handgrip strength were assessed. A single-slice computerized tomography image at the level of the umbilicus was acquired and the CSAs of IntraMAT, muscle tissue, and subcutaneous and visceral adipose tissues (SCAT and VAT) were calculated. The %IntraMAT significantly correlated with physical characteristics, such as age, BMI, abdominal circumference, and muscle tissue CSA. Stepwise multiple regression analysis was performed, with the %IntraMAT as the dependent variable. Muscle tissue CSA and abdominal circumference were the common independent variables across groups to explain the variability of the %IntraMAT. It was also extracted %body fat and age for Group 2, age, handgrip strength, and BMI for Group 3, and smoking category for Group 4. These results suggested that muscle tissue size and abdominal circumference would be the strong predictors to explain %IntraMAT of the trunk muscle across four groups, and that age, %body fat, BMI, and SCAT, handgrip strength and smoking category were also good predictors for each group with different exercise frequency in middle-aged Japanese men.

## Introduction

It is known that the regional distribution of adipose tissue plays a significant role in the development of metabolic and cardiovascular diseases [1–3], chronic inflammation [4], impaired glucose tolerance [5–8], and low strength and mobility [9–12]. There are several specific adipose tissue depots, including subcutaneous, visceral, and ectopic adipose depots, which include intramuscular, hepatic, and pancreatic fat depots. In particular, a great deal of attention has been paid to the role of intramuscular adipose tissue (IntraMAT). The accumulation of IntraMAT is recognized to be a result of aging, inactivity, spinal cord injury, obesity, and myopathy [1, 13, 14]. Although the roles of IntraMAT are not well known, it has been said to have a similar impact to visceral adipose tissue (VAT) with regard to the risk of metabolic defects, such as type 2 diabetes [15–17]. Furthermore, we have demonstrated that the cross-sectional area (CSA) of IntraMAT, determined using magnetic resonance imaging (MRI), is inversely related to muscle cross-sectional area (CSA) in both young and older individuals [18]. Similarly, Rossi et al. [19] showed that there was a moderate inverse correlation between IntraMAT and the size of the erector spinae muscles in 18 men and women aged 58–81 years (r = –0.79, P < 0.001), which implies that larger IntraMAT depots might lead to dysfunction in activities of daily living. These results suggest that IntraMAT has a negative impact on health [1].

Computed tomography (CT) imaging has been frequently used to quantify the size of muscles and adipose tissue depots using the Hounsfield Unit (HU) classified threshold [20–23]. Goodpaster et al. [21] showed that attenuation of the mid-thigh muscles on CT imaging moderately correlated with muscle fiber lipid content (r = –0.43, P < 0.01, n = 45) and triglyceride content (r = –0.58, P = 0.019, n = 19) in volunteers, which suggests that CT imaging can provide useful information regarding muscle and adipose tissue, including IntraMAT [21, 23].

It is obvious that body composition is closely related to the amount of physical activity performed. An increase in physical activity, in the forms of endurance and/or resistance training, is one of the most effective means of reducing IntraMAT and increasing muscle mass [24–27]. To obtain a sufficient training effect, it is important to appropriately customize the intensity, duration, and frequency of exercise. It may be easier to quantify the effects of training frequency on these parameters than the effects of differing duration or intensity of exercise. However, to our knowledge the effects of the frequency of exercise training on muscle and IntraMAT mass have not been well studied. With regard to the effect of resistance training frequency on skeletal muscle hypertrophy, however, Grgic et al. [28] showed that the frequency of resistance training does not have a positive effect on muscle mass. Willis et al. [29] determined the effect of the frequency of regular moderately intense exercise for 30 minutes on body fat in 90 unfit men and women, and found that a frequency of ≥4 times per week was associated with a significantly larger reduction in fat mass than a frequency of ≤3 times per week. However, further information regarding the effects of training frequency on IntraMAT and muscle are required, as well as how IntraMAT is related to demographic, morphologic, and functional parameters.

The purpose of this study was to examine how IntraMAT can be characterized using physical characteristics, muscle tissue CSA, and VAT or subcutaneous adipose tissue (SCAT) CSAs in participants with different exercise frequency in middle-aged Japanese men. We hypothesized that IntraMAT would be associated with muscle tissue CSA across groups with different exercise frequency, because there appears to be a robustness in the relationship between IntraMAT and muscle tissue CSA [18, 19].

## Materials and methods

### Participants

We performed a cross-sectional study of participants that were recruited for the Toyota Motor Corporation Physical Activity and Fitness Study (Aichi, Japan) between October 2015 and January 2016. One thousand twenty-eight middle-aged men were recruited. Before the study, the procedure, purposes, risks, and benefits associated with the study were explained and the written informed consent of all the participants was obtained. If the consent was waived before the measurement, the measurement was not performed, and if after the measurement, all data of the corresponding participants were deleted. The participants comprised skilled workers, clerical workers, and technicians and their specific work activities ranged from desk work to computer-aided design work, quality control, machining, and transportation. The participants were allocated to four groups, depending on the frequency with which they exercised each week, as recorded in a questionnaire. Group 1 comprised individuals who did not habitually exercise; Groups 2 and 3 comprised individuals who exercised once a week and twice a week or more, respectively; and Group 4 comprised individuals who exercised daily. The physical and demographic characteristics of the participants are shown in Table 1. The study was approved by the Director, Dr Yuji Yamamoto, of Institutional Review Board of the Research Center of Health, Physical Fitness & Sports at Nagoya University (number 28–15), and was conducted in accordance with the principles of the Declaration of Helsinki.

**Table 1:** Characteristics of participants.

		Group 1				Group 2				Group 3			Group 4		Effect size (partial η2)
Number of participants		334				271				269			154		
Age (years)	46.0	±	8.4	**	46.7	±	8.0	**	48.9	±	7.7	50.2	±	7.7	0.039
Height (cm)	170.8	±	5.9		170.2	±	6.0		170.3	±	5.5	170.3	±	5.7	0.002
Weight (kg)	67.1	±	9.7		67.4	±	9.1		67.3	±	9.1	65.7	±	9.2	0.004
BMI (kg/m^2^)	23.0	±	3.1		23.3	±	3.0		23.2	±	2.8	22.7	±	2.9	0.005
Body fat (%)	22.2	±	5.4	*	22.3	±	5.7	*	21.9	±	4.8	20.8	±	4.9	0.008
Abdominal circumference (cm)	81.3	±	8.5		81.2	±	8.4		80.7	±	8.0	79.7	±	8.1	0.005
Hypertension (n, %)		31, 9.3				39, 14.4				39, 14.5			15, 9.7		-
Diabetes (n, %)		11, 3.3				17, 6.3				21, 7.8			10, 6.5		-
Hyperlipemia (n, %)		35, 10.5				42, 15.5				45, 16.7			24, 15.6		-
Systolic blood pressure (mmHg)	117.7	±	14.2		118.1	±	13.6		119.4	±	14.1	116.6	±	12.9	0.004
Diastolic blood pressure (mmHg)	76.5	±	9.7		77.4	±	9.6		77.3	±	9.3	75.6	±	8.7	0.005
Handgrip strength (kg)	41.5	±	5.7		40.9	±	5.6		41.5	±	5.9	41.1	±	5.1	0.002
Smoking category (a.u.)	2.1	±	0.9		1.9	±	0.9		1.8	±	0.9	1.8	±	0.9	0.001
Drinking category (a.u.)	2.6	±	1.2		2.7	±	1.2		2.6	±	1.2	2.7	±	1.2	0.002
SCAT CSA (cm^2^)	154.7	±	65.0	*	153.5	±	64.6	*	147.3	±	58.6	137.3	±	56.6	0.021
VAT CSA (cm^2^)	62.4	±	42.0		64.0	±	45.3		65.3	±	45.6	54.9	±	36.4	0.006

Values are means and SD.

*, P < 0.05

**, P < 0.01 vs Group 4.

BMI, body mass index; CSA, cross-sectional area; SCAT, subcutaneous adipose tissue; VAT, visceral adipose tissue.

Group 1, no exercise habit; Group 2, exercise once a week; Group 3, exercise at least twice a week; Group 4, exercise every day.

Drinking category, category 1, no drinking; category 2, drink sometimes; category 3, 3 days a week; category 4, drink every day.

### Anthropometric measurements and blood pressure

Height, body mass and percentage body fat were measured using an automated body composition analyzer (BF-220, TANITA Corporation, Tokyo, Japan). Abdominal circumference was measured at the level of the umbilicus in the standing position at the end of expiration, while breathing normally. Resting blood pressure was measured using commercially available blood pressure measuring devices.

### CT imaging and image analysis

**Fig 1:**
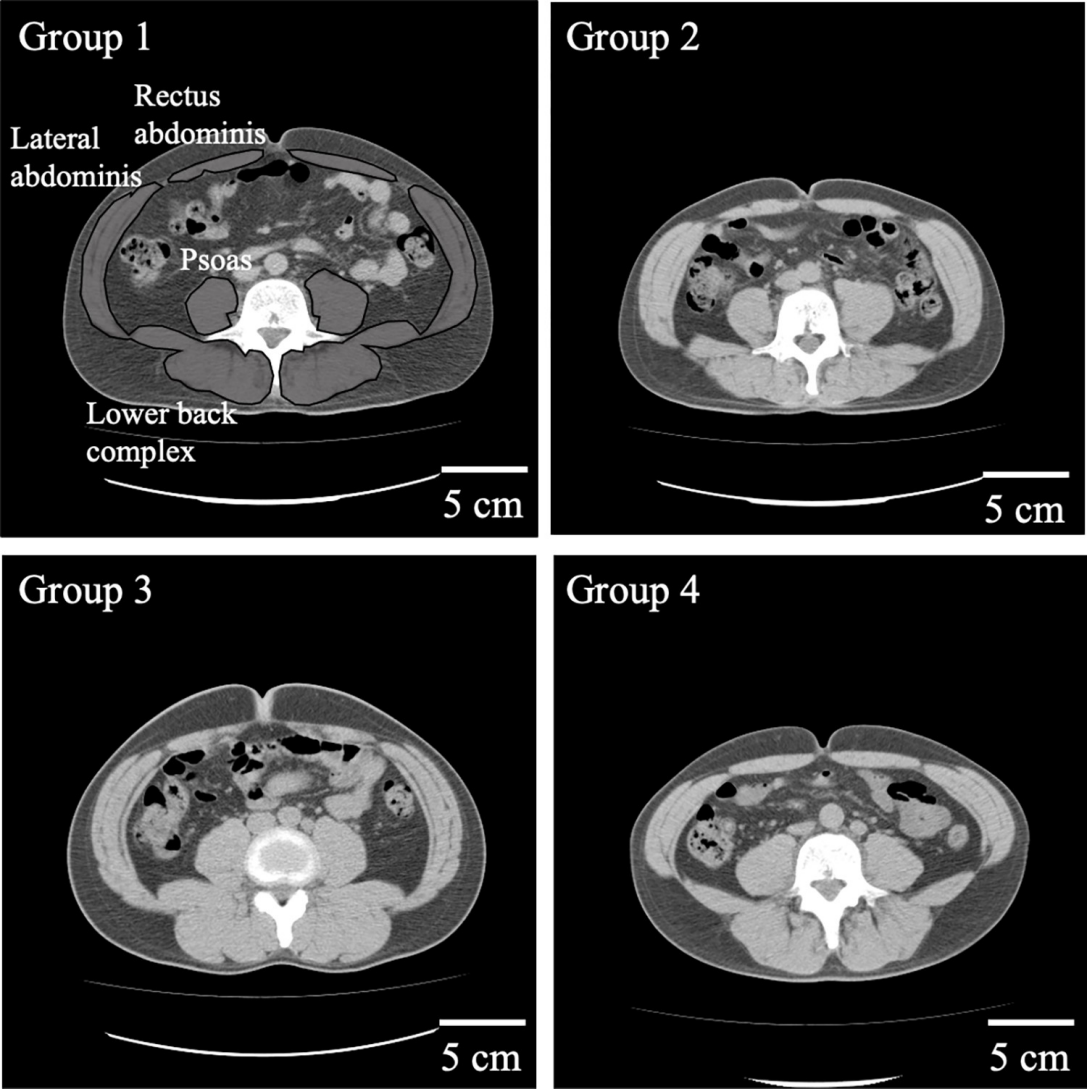
Representative computed tomography images of the trunk in Groups 1 to 4.

Transaxial images were obtained at the level of the umbilicus at the end of expiration using low-dose CT imaging (Toshiba Medical Systems, Tochigi, Japan), essentially as performed previously [30]. Representative images of participants in Groups 1–4 are shown in Fig 1.

The analysis in this study shows the results for the left and right muscles. Group 1 included individuals who has no exercise habit. Groups 2 and 3 were individuals with exercise habits once a week and twice a week or more, respectively. Group 4 consisted of individuals with exercise habit every day. Age of images of participants in Groups 1, 2, 3, and 4 were 47, 47, 48, and 51 years.

To analyze the CT images, the HU threshold for adipose tissue was calculated as the mean of three regions-of-interest (ROIs) located in the SCAT of each participant. The HU threshold for skeletal muscle was calculated as the mean of three ROIs located in the psoas muscle, because this muscle is known to have the lowest adipose tissue content of the muscles in the trunk region [20]. The muscle tissue CSA and %IntraMAT of the trunk (included rectus abdominis, lateral abdominis, psoas, and lower back complex) was calculated using the following equations:

Muscle tissue CSA (cm^2^) = (muscle CSA)—(IntraMAT CSA)

%IntraMAT = (IntraMAT CSA) / (muscle CSA) x 100

We also measured the CSAs of the VAT and SCAT. Image analysis was performed using the “region growing”, 2D, and morphology functions of SliceOmatic 5.0 software (TomoVision, Magog, Canada) by individuals who were blinded regarding the participant characteristics. The intra-class correlation coefficients for the analysis of each tissue were 0.999 for VAT, 0.938 for skeletal muscle, 0.855 for IntraMAT, and 0.999 for SCAT [31].

### Handgrip strength

Handgrip strength was measured using a pre-calibrated handgrip dynamometer (T.K.K. 5401, Takei Scientific Instruments, Niigata, Japan), with the participant standing upright. The maximum muscle strengths of the right and left hands were measured alternately, with a short interval between tasks, and the mean maximum force of each hand was used in subsequent analyses.

### Smoking and drinking habits

A self-administered questionnaire was used to quantify the participant’s smoking and drinking habits. Smoking habits were categorized as 1 for no smoking, 2 for previous smoking, and 3 for current smoking. Alcohol consumption habits were categorized as 1 for no alcohol consumption, 2 for occasional consumption, 3 for consumption on 3 days a week, and 4 for daily consumption.

### Statistical analyses

All values are reported as mean and standard deviation. One-way analysis of variance (ANOVA) was used to compare data for Group 4 with data for the other three groups. The partial η^2^ was calculated to evaluate the effect size among the groups. The Pearson product-moment correlation coefficient (r) was used to identify associations between variables. Forward stepwise multiple regression analysis was performed using variables that made a significant contribution to the variance (input criteria, P ≤ 0.05 and output criteria, P ≤ 0.100 were the default software settings). The dependent variable was the %IntraMAT of the trunk muscles and the independent variables were age, BMI, %body fat, abdominal circumference, diastolic blood pressure, systolic blood pressure, alcohol consumption category, smoking category, VAT and SCAT CSAs, and muscle tissue CSA and frequency of exercise (all groups analysis only), on the basis of the findings of our previous studies [18, 20, 32] and the results of simple correlation analyses. To avoid multicollinearity in the analysis, we checked that the variance inflation factor was lower than the set criterion of 10 in all the stepwise regression analyses. The level of significance was set at P < 0.05. All statistical analyses were performed using IBM SPSS Statistics for MacOS (version 26.0J; IBM, Inc., Armonk, NY, USA).

## Results

Table 1 shows the characteristics of the participants. One-way ANOVA revealed that there were significant effects of group in age (F_3,1024_ = 13.7, P = 0.001, partial η^2^ = 0.039); %body fat (F_3,1021_ = 2.78, P = 0.040, partial η^2^ = 0.008); SCAT (F_3,1024_ = 3.25, P = 0.021, partial η^2^ = 0.021).

Pairwise comparisons revealed that individuals in Group 4 were significantly older than those in Groups 1 (P = 0.001) and 2 (P = 0.001), the %body fat of Group 4 was significantly lower than those of Groups 1 (P = 0.025) and 2 (P = 0.020), and the SCAT CSA of Group 4 was significantly lower than those of Groups 1 (P = 0.011) and 2 (P = 0.024).

Fig 1 shows IntraMAT, muscle tissue CSAs, and %IntraMAT of the trunk muscles. One-way ANOVA revealed that there were significant effects of group in muscle tissue CSAs of the trunk muscles as a whole (F_3,1024_ = 3.68, P = 0.012, partial η^2^ = 0.011). Pairwise comparisons revealed that the muscle tissue CSA of the trunk muscles as a whole in Group 3 were significantly higher than those of Group 4 (P = 0.030).

Table 2 shows the correlation coefficients for the relationships between the %IntraMAT and other variables in Groups 1 to 4. The %IntraMATs significantly correlated with age (r = 0.336 to 0.393, all P = 0.001), weight (r = 0.167 to 0.253, P = 0.01 to 0.001), BMI (r = 0.185 to 0.357, P = 0.01 to 0.001), %body fat (r = 0.189 to 0.481, P = 0.01 to 0.001), and abdominal circumference (r = 0.284 to 0.428, all P = 0.001) in Groups 1 to 4. We also found significant correlation between %IntraMATs and systolic and/or diastolic blood pressure in Groups 1 to 3 (r = 0.118 to 0.195, P = 0.05 to 0.01), handgrip strength in Group 2 (r = -0.177, P = 0.01), smoking category in Group 4 (r = 0.259, P = 0.01), and drinking category in Groups 1 and 3 (r = 0.129, P = 0.05 and r = 0.156, P = 0.01). The %IntraMATs significantly correlated with SCAT (r = 0.245 to 0.381, all P = 0.001), VAT (0.288 to 0.474, all P = 0.001), and muscle tissue CSA (r = -0.452 to -0.532, all P = 0.001) in all groups.

**Table 2:** Correlation coefficients between %intramuscular adipose tissue (IntraMAT) and characteristics of participants in Groups 1 to 4.

			%IntraMAT of the trunk					
	Group 1		Group 2		Group 3		Group 4	
Physical characteristics								
Age	0.355	†	0.370	†	0.393	†	0.336	†
Weight	0.189	**	0.253	†	0.167	**	0.181	*
BMI	0.185	**	0.357	†	0.202	†	0.208	*
Body fat	0.189	**	0.481	†	0.302	†	0.326	†
Abdominal circumference	0.284	†	0.428	†	0.317	†	0.341	†
Blood pressure								
Systolic	0.118	*	0.181	**	0.195	**	0.088	
Diastolic	0.131	*	0.119		0.169	**	0.060	
Muscle strength								
Handgrip strength	-0.070		-0.177	**	-0.040		0.007	
Smoking and drinking status								
Smoking category	-0.005		-0.110		0.108		0.259	**
Drinking category	0.156	**	0.088		0.129	*	-0.096	
Adipose tissue CSA								
SCAT	0.245	†	0.381	†	0.296	†	0.331	†
VAT	0.288	†	0.474	†	0.386	†	0.406	†
Muscle tissue CSA	-0.452	†	-0.532	†	-0.461	†	-0.480	†

*, P < 0.05

**, P < 0.01

†, P < 0.001. IntraMAT, intramuscular adipose tissue.

Trunk includes the rectus abdominis, lateral abdominis, psoas, and lower back muscles.

BMI, body mass index; CSA, cross-sectional area; SCAT, subcutaneous adipose tissue; VAT, visceral adipose tissue. Group 1, no exercise habit; Group 2, exercise once a week; Group 3, exercise at least twice a week; Group 4, exercise every day.

Stepwise linear regression analysis was then performed using the %IntraMAT as the dependent variable and 17 independent variables for each group (Table 3). In all the groups, the muscle tissue CSA and abdominal circumference were found to be significant (Group 1, R = 0.655, P = 0.001; Group 2, R = 0.774, P = 0.001; Group 3, R = 0.687, P = 0.001; Group 4, R = 0.695, P = 0.001). In addition, the analysis showed that %body fat and age were significant variables in Group 2; age, handgrip strength, and BMI in Group 3; and smoking habits in Group 4.

**Table 3:** Stepwise linear regression analysis as a dependent variable of %IntraMAT of the trunk muscle.

Group	Dependent variables	Independent variables	Regression coefficient	SE	Standardized regression coefficients	VIF	P	R	Adjusted R^2^
Group 1	%IntraMAT of the trunk	Muscle tissue CSA	-0.207	0.015	-0.634	1.152	0.001	0.655	0.425
		Abdominal circumference	0.383	0.034	0.513	1.152	0.001		
Group 2	%IntraMAT of the trunk	Muscle tissue CSA	-0.205	0.016	-0.561	1.287	0.001	0.774	0.593
		Abdominal circumference	0.265	0.056	0.310	2.809	0.001		
		%body fat	0.331	0.078	0.264	2.556	0.001		
		Age	0.099	0.039	0.111	1.275	0.012		
Group 3	%IntraMAT of the trunk	Muscle tissue CSA	-0.183	0.016	-0.607	1.451	0.001	0.687	0.461
		Abdominal circumference	0.173	0.077	0.212	4.457	0.026		
		Age	0.179	0.042	0.212	1.208	0.001		
		Handgrip strength	0.165	0.055	0.150	1.233	0.003		
		BMI	0.508	0.224	0.222	4.773	0.024		
Group 4	%IntraMAT of the trunk	Muscle tissue CSA	-0.188	0.020	-0.591	1.096	0.001	0.695	0.473
		Abdominal circumference	0.383	0.049	0.479	1.100	0.001		
		Smoking category	1.010	0.434	0.139	1.036	0.021		
All groups	%IntraMAT of the trunk	Visceral adipose tissue CSA	0.026	0.006	0.169	2.212	0.001	0.529	0.276
		Age	0.276	0.024	0.340	1.188	0.001		
		%body fat	0.357	0.067	0.285	3.971	0.001		
		BMI	-0.811	0.125	-0.366	4.471	0.001		
		SCAT	0.030	0.006	0.284	4.201	0.001		
		Smoking category	0.391	0.194	0.054	1.015	0.044		

BMI, body mass index; CSA, cross-sectional area; IntraMAT, intramuscular adipose tissue; SE, standard error; VIF, variance inflation factor.

Independent variables: abdominal circumference, age, BMI, %body fat, diastolic blood pressure, systolic blood pressure, alcohol consumption category, smoking category, visceral adipose tissue CSA, subcutaneous adipose tissue CSA, and muscle tissue CSA of the trunk.

Note for analysis of All groups: Since muscle tissue CSA and abdominal circumference were selected in the analysis of all groups. Then, muscle tissue CSA and abdominal circumference were excluded from the independent variables in the analysis for all groups to find other independent variables that may explain the %IntraMAT of the trunk. Also, exercise frequency was added as an independent variable.

By excluding muscle tissue CSA and abdominal circumference from the independent variables which were found to have a strong influence on %IntraMAT and adding the frequency of exercises, another stepwise multiple linear regression analysis was performed on all participants to find factors that could better explain the %IntraMAT (Table 3). Then, visceral adipose tissue CSA, age, %body fat, BMI, SCAT, and smoking category were extracted to explain the variance of the %IntraMAT.

## Discussion

**Fig 2:**
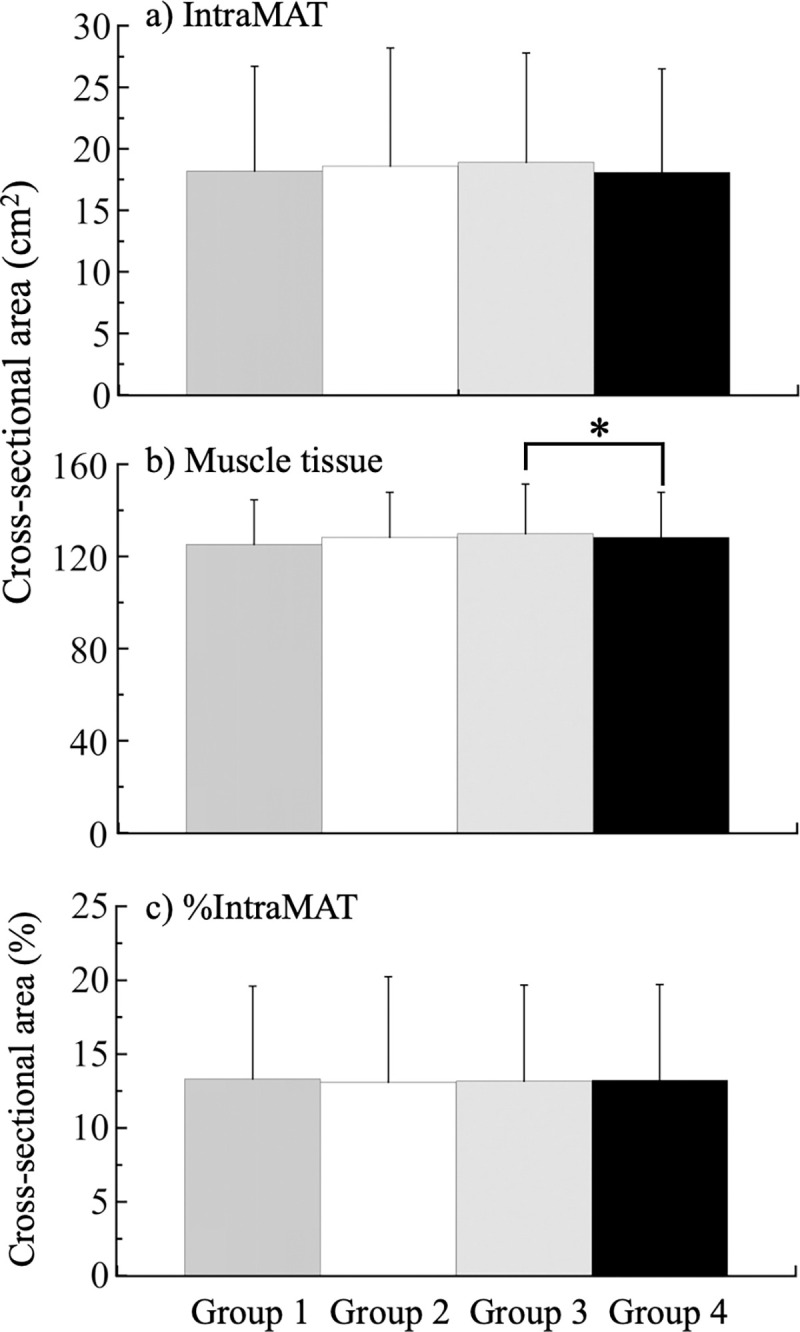
Cross-sectional areas of intramuscular adipose tissue (IntraMAT), muscle tissue, and %IntraMAT of Groups 1 to 4. *, P < 0.05.

The purpose of this study was to examine how the IntraMAT can be characterized using physical characteristics, muscle tissue CSA, VAT and SCAT CSAs, and handgrip strength in participants with different exercise frequency in middle-aged Japanese men. Contrary to our expectations, the difference in the frequency of exercise did not show up clearly in the IntraMAT CSA, muscle tissue CSA, or %IntraMAT among the exercise groups (Fig 2). However, the %IntraMAT of each group significantly correlated with physical characteristics, including age and abdominal circumference (Table 2). In particular, the %IntraMAT of the trunk muscles significantly correlated with the physical characteristics of the participants in all the exercise groups. Furthermore, stepwise linear regression analysis revealed that muscle tissue CSA and abdominal circumference were significantly and independently associated with IntraMAT across the groups.

There were significant differences in some parameters of physical characteristics among four groups. These points should be noted when considering the differences in parameters between the groups shown below. The %body fat and SCAT CSA of Group 4 were significantly lower than those of Groups 1 and 2, but not of Group 3 (Table 1). This finding was predictable, because it would be expected that lower exercise frequency could lead to greater fat deposition. Another interesting finding was that the mean age of Group 4 was significantly higher than those of Groups 1 and 2, implying that age and the frequency of exercise were inversely related.

No statistical processing has been performed, but we found that almost all the correlation coefficients for the relationships between %IntraMAT and VAT CSA appeared to be similar to those for the relationship between %IntraMAT and SCAT CSA in all the groups (Table 2). A similar result was shown by Boettcher et al. [15] who showed that correlation coefficients between IntraMAT (IMAT was used in their study) and SCAT or VAT were both 0.42 (P < 0.0001) in 97 men aged 48.1 ± 11.4 years. Furthermore, Song et al. [33] demonstrated that a parallel increase in both IMAT and VAT in weight-stable women during a two-year period. These results suggested the there is a linear relationship between changes in VAT and SCAT CSAs, suggesting that the patterns of change in the two parameters could be similar.

One of the most important findings of the present study was that IntraMAT and muscle tissue CSA inversely correlated in all groups (Table 2). This finding is consistent with that of a previous study [18], which showed that the %IntraMAT was inversely related to the muscle tissue CSA of the quadriceps femoris, regardless of age (Young, r = –0.586, P < 0.05; Older, r = –0.672, P < 0.01). Rossi et al. [19] also showed that IntraMAT (the original was described as IMAT) was inversely related to the CSAs of the erector spinae muscles using magnetic resonance imaging in 18 healthy men and women with mean age of 70.1 ± 8.0 years, and importantly, that the relative IMAT to total area was inversely associated with muscle CSA (r = -0.79, P < 0.001). The participants in this study were middle-aged men and those in Rossi et al.’s [19] study were elderly, direct comparison of these results should be made with caution. However, as mentioned earlier, it is also reported that a significant correlation between IntraMAT and muscle CSA was observed in both young and elderly participants in our study (15 young, r = –0.586, P < 0.05; 15 elderly, r = –0.672, P < 0.01) [18]. The findings of these previous studies are highly consistent with the relationship between %IntraMAT and muscle tissue CSA identified in the present study.

What we should note is that there are racial difference in the IntraMAT accumulation. Gallager et al. [34] showed that African Americans had a significantly greater increment in IntraMAT (intermuscular adipose tissue (IMAT) was used in their paper) of the whole body compared with Whites and Asians using MRI. Miljkovic et al. [35] also showed IMAT in 518 Afro-Caribbean men were significantly higher than 1105 Caucasian men with no significant difference in total body fat. These results suggest that there seemed to been race-related difference in IntraMAT; however, all participants were Japanese in this study, therefore, we should not take into account of race-related difference in %IntraMAT in this study.

Stepwise linear regression analysis for each group, using the %IntraMAT of the trunk muscles as the dependent variable, revealed relationships with muscle tissue CSA and abdominal circumference for Group 1 (adjusted R^2^ = 0.425, P = 0.001); muscle tissue CSA, abdominal circumference, and %body fat for Group 2 (adjusted R^2^ = 0.593, P = 0.001); muscle tissue CSA, abdominal circumference, age, handgrip strength, and BMI for Group 3 (adjusted R^2^ = 0.461, P = 0.001); and muscle tissue CSA, abdominal circumference, and smoking habits for Group 4 (adjusted R^2^ = 0.473, P = 0.001). Among the identified independent variables, muscle tissue CSA and abdominal circumference were common for all the groups, which implies that these variables can explain the %IntraMAT of each group, and furthermore, the absolute standardized regression coefficients for the muscle tissue CSA were the highest, followed by those for abdominal circumference.

We also found there were specific selected variables for each group. For example, %body fat was specific for Group 2, age was selected for Groups 2 and 3, BMI and handgrip strength for Group 3, and smoking category was for Group 4. The parameters chosen for each group appear to be reasonable, because most of the group-specific variables significantly correlated with %IntraMAT in Table 2. Among them, handgrip strength was extracted to explain %IntraMAT in Group 3, which was unique because no significant correlation in simple linear analysis in Table 2 (r = -0.040, n.s.). Furthermore, handgrip strength was positively correlated to %IntraMAT, conflicting to a significant relationship between handgrip strength and %IntraMAT in Group 2 (r = -0.177, P < 0.01). The reasons for the results cannot be fully explained. However, the lack of significant correlation with %IntraMAT in the single correlation and the lower tendency of standardized regression coefficients compared to other significant independent variables (e.g. age or BMI) suggest that the relationship may be not very close. Further study in this regard will be necessary.

The finding that muscle tissue CSA was able to at least in part explain the %IntraMAT of the trunk muscles across the groups is consistent with the finding of our previous study of 30 young and older individuals [18] that the %IntraMAT could be explained by the muscle tissue CSA of the thigh, measured using MRI (young, adjusted R^2^ = 0.410; older, adjusted R^2^ = 0.502). In addition, the adjusted R^2^ values in the present study (Table 3, adjusted R^2^ = 0.425–0.593) were similar to those obtained in our previous study, as shown above, which suggests that 40%–50% of the variance in %IntraMAT may be explained by the muscle tissue CSA, irrespective of the age of the individuals. Furthermore, the same relationship between IntraMAT and muscle tissue CSA was identified in the trunk and thigh muscles, suggesting that this relationship may exist in multiple anatomic locations.

The other common independent variable that may explain the %IntraMAT was abdominal circumference. Abdominal circumference is a simple and easy method of estimating VAT CSA [36]. Although there were no significant differences in abdominal circumference or VAT CSA among the exercise groups in the present study, significant relationships between the %IntraMAT of the trunk muscles as a whole and abdominal circumference were found in all four groups, with R-values ranging from 0.284 to 0.428. These R-values were very similar to those for the relationships between the %IntraMAT of the trunk muscles as a whole and the VAT CSA for each group, which ranged from 0.288 to 0.474. It was reported that the amounts of both IntraMAT and VAT are linearly related with the adipose tissue mass of the whole body [34], which may explain why VAT was found to be independently associated.

Since muscle tissue CSA and abdominal circumference were found to have a strong influence on %IntraMAT in each group, stepwise multiple regression analysis was performed on all subjects without including these two factors as independent variables. As a result, two previously non-extracted variables, i.e. VAT CSA and SCAT, and four previously selected variables, i.e. age, %body fat, and BMI, were extracted. This result seemed reasonable, because both VAT CSA and SCAT were significantly correlated with %IntraMAT in all groups in Table 2. This result supports Gallagher et al. [34] who demonstrated that total adipose tissue of the body was significantly correlated with VAT and IntraMAT. Thus, it is expectable that VAT CSA, %body fat, BMI and SCAT, which are recognized as indices of body fat, were extracted as independent variables. Among these independent variables, BMI was negatively associated with %IntraMAT (standardized regression coefficient, -0.366, P < 0.001). This relationship was unexpected because %IntraMAT and BMI was positively correlated in Group 3 in Table 3 and simple correlation analysis all four groups as shown in Table 2. The reason of the result was not explained well in this study. BMI itself was negatively related to %IntraMAT, but other confounding factors may be involved, but it is unclear from this study.

Finally, the participants’ smoking habits were also found to, at least in part, explain the %IntraMAT of Group 4. Terry et al. [37] found that the abdominal IntraMAT volume of people who had never smoked (2.25 cm^3^) was significantly lower than those of former smokers (2.42 cm^3^) and current smokers (2.51 cm^3^) in their study of 3,010 middle-aged men and women. However, the difference in the IntraMAT among the three groups was very small (≤ 0.26 cm^3^). In the present study, there were no significant differences in IntraMAT among the four groups (Fig 2), and we are unable to explain why smoking habits were identified as an independent variable in Group 4. Future studies may explain this finding.

The limitation of this study was we asked frequency of exercise a week using a questioner, therefore, we do not have any information on intensity, duration and types of exercise. This information may be more important parameters than frequency of exercise a week. We believe that these points can be explored in future studies.

In conclusion, we have determined the effect of exercise frequency on the relationships of %IntraMAT with body composition, handgrip strength and muscle tissue CSA in 1,028 middle-aged men. Stepwise multiple linear regression analysis revealed that muscle tissue CSA and abdominal circumference were independent variables that could explain the variability in the %IntraMAT of the trunk muscles. These results suggested that muscle size and abdominal circumference would be the strong predictors to explain %IntraMAT of the trunk muscle across four groups, and that age, %body fat, BMI, and SCAT, handgrip strength and smoking category were also good predictors for each group with different exercise frequency in middle-aged Japanese men.
